# Combinatorial gene therapy for epilepsy: Gene sequence positioning and AAV serotype influence expression and inhibitory effect on seizures

**DOI:** 10.1038/s41434-023-00399-w

**Published:** 2023-04-07

**Authors:** Esbjörn Melin, My Andersson, Casper R. Gøtzsche, Jenny Wickham, Yuzhe Huang, Julia Alicja Szczygiel, Arnie Boender, Søren H. Christiansen, Lars Pinborg, David P. D. Woldbye, Merab Kokaia

**Affiliations:** 1 Experimental Epilepsy Group, Epilepsy Centre, Lund University Hospital, 17 Sölvegatan, 221 84 Lund, Sweden; 2 CombiGene AB, Medicon Village, 2 Scheelevägen, 223 81 Lund, Sweden; 3 Department of Neuroscience, Panum Institute, University of Copenhagen, 3B Blegdamsvej, DK-2200 Copenhagen N, Denmark; 4 Department of Neurology and Neurobiology Research Unit, Copenhagen University Hospital, Rigshospitalet, 9 Blegdamsvej, DK-2100 Copenhagen, Denmark

**Keywords:** Regeneration and repair in the nervous system, Neurological disorders

## Abstract

Gene therapy with AAV vectors carrying genes for neuropeptide Y and its receptor Y2 has been shown to inhibit seizures in multiple animal models of epilepsy. It is however unknown how the AAV serotype or the sequence order of these two transgenes in the expression cassette affects the actual parenchymal gene expression levels and the seizure-suppressant efficacy. To address these questions, we compared three viral vector serotypes (AAV1, AAV2 and AAV8) and two transgene sequence orders (NPY-IRES-Y2 and Y2-IRES-NPY) in a rat model of acutely induced seizures. Wistar male rats were injected bilaterally with viral vectors and 3 weeks later acute seizures were induced by a subcutaneous injection of kainate. The latency until 1^st^ motor seizure, time spent in motor seizure and latency to status epilepticus were measured to evaluate the seizure-suppressing efficacy of these vectors compared to an empty cassette control vector. Based on the results, the effect of the AAV1-NPY-IRES-Y2 vector was further investigated by in vitro electrophysiology, and its ability to achieve transgene overexpression in resected human hippocampal tissue was evaluated. The AAV1-NPY-IRES-Y2 proved to be better to any other serotype or gene sequence considering both transgene expression and ability to suppress induced seizures in rats. The vector also demonstrated transgene-induced decrease of glutamate release from excitatory neuron terminals and significantly increased both NPY and Y2 expression in resected human hippocampal tissue from patients with drug-resistant temporal lobe epilepsy. These results validate the feasibility of NPY/Y2 receptor gene therapy as a therapeutic opportunity in focal epilepsies.

## Introduction

Epilepsy is a common neurological disorder that is characterized by recurrent episodes of synchronous neuronal hyperactivity leading to seizures that are often associated with behavioural symptoms as convulsions. It is estimated that approximately 1% of the population suffers from epilepsy [1, 2] and that the related yearly costs for the disorder in Europe was 15.5 billion euros in 2004 [3]. Anti-seizure drugs (ASDs) are helping most of the patients, but one third of the cases are drug-resistant [4]. Many of those suffering from intractable epilepsy have temporal lobe epilepsy (TLE), where the seizure focus is predominately located in the hippocampus or amygdala [5]. A majority of these cases (approximately 2/3) are drug-resistant [4]. There are few alternatives to ASDs, e.g., surgery, vagus nerve stimulation (VNS) [6], or ketogenic (high fat) diet [7]. Although effective [8], surgical resection of the temporal lobe and other epileptic foci is not always applicable [1], while VNS and ketogenic diet has variable efficacy. Therefore, novel treatments are urgently needed, and gene therapy emerges as a strong alternative approach [9].

Neuropeptide Y (NPY) is a 36 amino acid peptide that is expressed in the human brain [10] predominantly by inhibitory interneurons [11] and is released in response to high frequency activity [12]. NPY is primarily interacting with three G-protein coupled receptors in the brain, Y1, Y2, and Y5 [13]. The Y2 receptor has been shown to localize on presynaptic terminals of excitatory neurons and inhibit glutamate release by reducing opening of voltage gated Ca^+^-channels [14]. Interestingly, in hippocampal tissue resected from epilepsy patients, NPY and the Y2 receptors are upregulated, suggesting that it may be an endogenous response to counteract hyper-excitability [15].

The antiseizure properties of NPY, mediated by interaction with the Y2 receptors, has been described in number of previous studies [16–21] (see also [22, 23]). We have previously shown that adeno-associated viral (AAV) vector-mediated overexpression of NPY and the Y2 receptors suppresses acutely induced seizures in two in-vivo animal models [24], as well as chronic spontaneous recurrent seizures (SRS) in the rat intrahippocampal kainate (KA) model of TLE [25, 26]. Based on the accumulating evidence, combinatorial NPY and Y2 receptor overexpression by AAV vectors could be a candidate for gene therapy against focal epilepsies [23].

It is not known, however, how different AAV vector serotypes on one hand, and sequential order of multiple transgenes in the expression cassette on the other hand affects the magnitude of the seizure-suppressant efficacy of this treatment. The AAV1, AAV2 and AAV8 serotypes have been reported to achieve effective delivery of transgenes into the hippocampal cells [27]. However, the various AAV serotype capsids have been shown to have different tropisms, e.g., in distinct hippocampal areas [28], which might affect functional outcome. Also, the internal ribosome entry site (IRES) sequence separating two gene sequences, e.g. for the NPY and the Y2 receptor, is reported to reduce translation of the downstream gene [29], which also may have implications for the potential anti-seizure effect. Thus, in the present study we compared the efficacy of NPY and its receptor Y2 overexpression delivered by three different viral vector serotypes, each with either the NPY-IRES-Y2 or Y2-IRES-NPY sequence order of the genes.

We found that the AAV1 serotype with a gene sequence of NPY-IRES-Y2 (AAV1-NPY/Y2) had a significant pharmacodynamic effect by reducing time spent in motor seizures and increasing the latency to status epilepticus (SE) induced by systemic KA in male Wistar rats. This vector also showed a clear overexpression of NPY as evaluated by immunoreactivity, as well as increased functional binding of NPY to Y2-receptors compared to control animals injected with empty vector. In line with findings of inhibitory effects on seizures in vivo, electrophysiological analysis in acute hippocampal slices from AAV1-NPY/Y2 injected animals revealed decreased glutamate release probability in synapses at high frequency activity. Finally, we demonstrate that AAV1-NPY/Y2 vector can well express respective transgenes in human hippocampal slices in vitro, corroborating translational value of this approach.

## Method

### Animals

Male Wistar rats (Charles River, Denmark), weighing 220–350 g at beginning of the experiment, were housed together with two or three animals in each cage. The animals had *ad libitum* access to food and water, fixed 12 h day/light cycle and the cages were enriched by a cardboard tunnel and nesting materials. After arrival to the facilities, the rats were left in the stables for at least 1 week before they were subjected to any operations in order to enable acclimatization to the new environment. All experiments were approved by the Malmö/Lund Ethical Committee for Experimental Animals (Permit number M49-15) and performed according to international guidelines for the use of research animals.

A total number of 77 rats were used in the KA induced seizure test and randomly divided into 9 treatment groups by an experimenter not aware of the treatment conditions. The resulting group size was 8 in all groups, except for increased numbers of rats administered AAV8-NPY/Y2 (*n* = 12) and AAV8-Y2/NPY (*n* = 9), since 5 rats were added in these groups due to lost data at video recordings that were later recovered. 12 rats were used in the electrophysiology test (*n* = 4/group). The group sizes were by experience considered appropriate for statistical comparison.

### Viral vectors

Viral vectors were manufactured by GeneDetect (New Zealand) and provided by CombiGene AB (Sweden). A set of cDNA constructs, with a size of 4.3kbp, carrying the sequence for human NPY and the human NPY Y2-receptor, separated by an internal ribosome entry site (IRES), with either NPY first (NPY/Y2) or the Y2-receptor first (Y2/NPY), were contained in three different AAV-serotypes, AAV1, AAV2 and AAV8. The vectors all contained the Woodchuck post-transcriptional regulatory element (WPRE) and bovine growth hormone polyadenylation sequence (BGHpA). Also, three empty control vectors (EMPTY) were used, one for each serotype, resulting in a total number of nine vectors for evaluation. The synthetic CAG (chicken-beta-acting promoter hybridized with the cytomegalovirus [CMV] immediate-early enhancer sequence) were driving the constructs. All viral vectors were titrated together using qPCR targeting the WPRE and had a titer of 1.1*10^12^ viral genomes (vg)/ml.

### Viral vector delivery

The viral vectors were delivered bilaterally to the hippocampus via stereotaxic surgery. Rats were anesthetized with 4% isoflurane (Isoba Vet, Intervet, Netherlands) air mixture lowered to 2% when paw withdrawal response was absent. The rat’s heads were shaved and placed in a stereotaxic frame (Kopf Instruments, Tujuga, CA, USA). Local anaesthesia (Marcain, AstraZeneca, Denmark) was administered before an incision was made exposing the scull bone. Drops of hydrogen peroxide (4%) were applied to stop bleeding after scalpel scrapes and to expose bregma. Holes were drilled, prior to each injection, using predefined coordinates (see below). The viral vector, kept on ice at all time before the injection, was loaded into a glass capillary attached to a microliter syringe (Hamilton Company, Switzerland) and delivered into the hippocampus at a rate of 0.2 µl/min. Dorsal hippocampus (AP −3.3 mm. ML ± 1.8 mm, DV −2.6 mm) was injected with 1.0 µl vector and ventral hippocampus (AP −4.8 mm, ML ± 5.2 mm) was injected with 1.0 µl at two different depths (DV −6.4 and −3.8) [30]. Thus, a total of 6 µl viral vector at a concentration of 1.1*10^12^ vg/ml were delivered to each brain, resulting in a dose of 6.6*10^9^ vg/brain. Reference points were bregma for anterior/posterior coordinate, midline for medial/lateral and dura for dorsal/ventral. The tooth bar was set to -3.3 mm. After vector delivery, the incision was closed using staples, and the animals were allowed to recover in quarantine for 48 h before being relocated to their stables.

### Controlling for orexigenic effects of NPY and Y2 overexpression

As NPY is an established orexigenic factor [31–33], we were interested to study if the different vectors had any effect on animal weight development. No difference in weight gain per day was detected between the groups (Supplementary Fig. 1).

### KA preparation and induction of SE

KA was prepared freshly the same day as SE induction. 50 mg of KA (Abcam, ab120100) was dissolved in 4 ml of saline and adjusted with 1 M NaOH to a pH of 7.4, in order to reduce a pain response in connection to the injection. The concentration was set to 10 mg/ml by adding saline to a total volume of 5 ml. Continuous measurement of pH during NaOH titration and comparing to previous titration curves facilitated reproducibility. The rats were injected with a dose of 10 mg/kg in the neck region subcutaneously (s.c.) and immediately placed in a transparent Plexiglas cage with the dimension 30 x 19 x 29 cm. Ten animals per time were treated with KA and recorded for 2 h with a high-definition camera.

### Assessment of KA-induced seizures

The animals were assessed for behavioral manifestation of seizures for two hours following KA injection. Behavioral scoring of seizures was performed according to a modified Racine scale [24, 34], focusing exclusively on grade 3–5 seizures. Grade 3 seizures included a minimum of 15 s of clonic activity in the forelimbs, in a grade 4 seizure the rat was rearing while the clonus in the forelimb continued and a grade 5 seizure caused the animal to rear and fall on side or back. The analysis of the video recordings was performed by an experienced experimenter blinded to the treatment conditions. Three parameters were assessed. First, latency to first motor seizure was measured as the time that passed from the injection until the 1^st^ grade 3 seizure. Second, the time spent in motor seizures (grade 3 or above) was measured. Again, a seizure had to be at least 15 s to add to the cumulative seizure duration. Finally, the time elapsed from injection until SE, defined as continuous clonic activity of at least 10 min, was measured. The monitoring was terminated after 2 h by deeply anesthetizing the animals with isoflurane and decapitation.

### Immunohistochemistry and assessment of NPY expression levels

The brains were quickly removed from the skull, directly frozen on crushed dry ice and stored at −80 **°**C until being cut in 16 µm thick coronal slices utilizing a cryostat. The resulting 16 series of slices, covering the dorsal and ventral injection points, were also stored in −80 **°**C on glass slides until further processing.

Before immunohistochemical staining, the slices were fixed by soaking the glass slides with slices in 4% paraformaldehyde (PFA) for 20 min. After washing in 0.02 M KPBS, the tissue was pre-incubated in 10% donkey serum in 0.25% T-KPBS for 1 h followed by primary antibody (Rabbit anti-NPY, N9528, Sigma Aldrich, 1:500) incubation overnight at 4 **°**C in 5% donkey serum and 0.25% T-KPBS. Next day the slices were washed and then incubated with secondary antibody (Cy3 conjugated Donkey anti Rabbit, Jackson Immunoresearch, 1:200) in 1% donkey serum, 0.25% T-KPBS solution for 2 h at room temperature. After washing a final time in 0.2 M KPBS the slices were cover-slipped in DABCO (Sigma Aldrich). A negative control (primary antibody omitted) was used to control for unspecific binding of the secondary antibody.

For analysis, 6 fluorescent microscopy pictures (Olympus BX61) were taken from each animal. Two of the pictures covered the dorsal injection points and four the ventral hippocampus. Settings for image acquisition were the same for all images (ISO800, 1500 ms exposure, 4x objective). The mean gray value of the fluorescence in the hippocampus was measured by ImageJ and was corrected for by the background level in a cortical region were no NPY overexpression was observed (see representative images of NPY immunohistochemistry in the dorsal hippocampus in Supplementary Fig. 2).

### Y2 receptor functional binding

Visualization of Y2 functional binding has been described earlier [24, 35, 36]. The tissue sections were defrosted in room temperature for 30 min before rehydration in assay buffer A (50 mM Tris-HCl, 3 mM MgCl, 0.2 mM ethylene glycol tetraacetic acid [EGTA], 100 mM NaCl, pH 7.4) for 10 min at room temperature. Thereafter, a pre-incubation assay buffer B (assay buffer A + 0.2 mM dithiothreitol, 1 µM 1,3-dipropyl-8-cyclopentylxanthine [DPCPX; #C-101, Sigma Aldrich, DK], 0.5% w/v bovine serum albumin, 2 mM guanocine-5’-diphosphate [GDP; #G7127, Sigma Aldrich]) was applied for 20 min at room temperature. Subsequently, the sections were incubated in assay buffer C (assay buffer B + 40 pM [^35^S]-GTPγS [1250 Ci/mmol; NEG030H250UC; PerkinElmer, DK]) together with 1 µM NPY (rat synthetic, Schafer-N, Copenhagen, DK) and the Y1 (1 µM BIBP3226, #E3620, Bachem AG, Switzerland) and Y5 (10 µM L-152,804, #1382, Tocris Cookson, UK) antagonists for 1 h at 25 °C. The assay was terminated by rinsing the sections 2 × 5 min in ice-cold 50 mM Tris-HCl buffer (pH 7.4) and subsequently dried. Exposure to Kodak BioMax MR films was done together with ^14^C micro scales (Amersham Life Sciences, UK) during 5 days in −20 °C and the films were thereafter developed in a Kodak GBX developer.

The NPY stimulated binding was analysed by measuring the mean grey value of the hippocampus. The value from the hippocampus was corrected for by the background grey value in a cortical region were no Y2 overexpression was observed (see representative image of Y2 receptor functional binding in Supplementary Fig. 3).

### Preparation of acute hippocampal slices

Preparation of acute slices was performed as previously described [37]. Animals were sacrificed by decapitation under isoflurane anaesthesia and the brain was quickly removed, with the rAAV-injected hemisphere cut transversely on a vibratome in sucrose-containing artificial cerebrospinal fluid (sucrose-aCSF), containing in (mM) 75 sucrose, 67 NaCl, 26 NaHCO_3_, 25 glucose, 2.5 KCl, 1.25 NaH_2_PO_4_, 0.5 CaCl_2,_ and 7 MgCl_2_. Hippocampal slices (400 µm) were collected and placed in a holding chamber with aCSF containing (in mM): 119 NaCl, 2.5 KCl, 1.3 MgSO_4_, 26.2 NaHCO_3_, 1 NaH_2_PO_4_, 11 glucose and 2.5 CaCl_2_, oxygenated at room temperature (RT), and left to rest for approximately 1 h. For recordings, slices were transferred to the recording chamber perfused with oxygenated aCSF (RT; 2 ml/min).

### Electrophysiology

Field recordings of excitatory postsynaptic potentials (fEPSPs) were conducted in the stratum radiatum of hippocampal CA1 region. Schaffer collaterals were stimulated with square constant current pulses (0.1 µs) through silver-chloride electrodes in glass capillaries filled with aCSF. Field EPSPs were recorded from the same layer with a recording capillary filled with aCSF (with a pipette resistance of 1–3 MΩ). Each individual slice was considered as one *n*. Field EPSP recordings were started by determining the input-output relationship between the amplitude of the presynaptic fiber volley (PSFV) and the amplitude of the fEPSP. Only slices capable of generating fEPSP amplitudes of more than 1 mV were included in the study. Test stimuli inducing 30–50% of the maximal fEPSP responses were used throughout all experiments. Short-term plasticity of fEPSPs was assessed by paired-pulse (PP) stimulations at different interstimulus intervals (ISI; 25, 50, 100, 200 ms; at 0.033 Hz). Paired-pulse ratio (PPR) of fEPSPs was calculated as change in the initial slope of the second fEPSP as compared to the first.

The effect of transgene NPY expression on excitatory neurotransmission during repetitive synaptic activation was examined by applying high-frequency stimulation (HFS) trains of 100 Hz with 10 stimulations of one synaptic pathway (SP.1) preceded and followed with 500 ms interval by PP-stimulation in a neighboring, convergent but independent synaptic pathway (SP.2). Synaptic independence was determined by evoking a fEPSP in SP2, with or without a fEPSP 50 ms prior in SP.1. The lack of paired-pulse facilitation supports the absence of shared synapses between the inputs. The stimulation was repeated eight times and the responses were averaged. To avoid interference with post-tetanic potentiation (PTP), each train was delivered at 5 min intervals. To prevent long-term potentiation (LTP) induction in stimulated synapses, the specific N-methyl-D-aspartate (NMDA) antagonist, D(2)-2-amino-5-phosphonopentanoic acid (D-AP5, 50 µM) was applied together with aCSF. The specific Y2-receptor antagonist BIIE0246 (0.6 μM, Sigma-Aldrich) was applied to the aCSF and allowed to wash in during paired-pulse stimulation for 10 min. After recordings, slices were fixed in PFA (12 h at 4 °C), rinsed 3 × 20 min in KPBS and stored in Walters antifreeze solution (−20 °C).

### Immunohistochemistry in acute hippocampal slices

Sections were washed three times in KPBS for 10 min and imbedded in a solution containing 300 g/L egg-albumin (Sigma-Aldrich) and 30 g/L gelatin (Sigma-Aldrich), frozen at −80 °C and sub-sliced on a cryotome at −20 °C (Cellab Nordia AB) to 16 μm thick slices. Slices were mounted on positively charged glasses (+Menzel glass, Thermo Scientific) and stored at −20 °C. These slices were then preincubated in 10% Donkey serum, 0.25% Triton X-100 in KPBS for 1 h. After adding rabbit anti-NPY antibody (1:500, #N9528, Sigma-Aldrich, DK) in 5% Donkey serum, 0.25% Triton X-100 in KPBS, sections were incubated overnight at 4 °C. Sections were washed 3 × 10 min in tKPBS, incubated for 2 h at RT with Cy3-conjugated Donkey anti-rabbit antibody (1:200, Jackson Immunoresearch USA) in 1% Donkey serum, 0.25% Triton X-100 in KPBS and washed 1 × 10 min in tKPBS and 2 × 10 min in KPBS. The slides were mounted with DABCO (Sigma-Aldrich) and digitized images obtained using Olympus BX61 microscope and CellSens software.

### Human organotypic slice preparation and AAV-vector mediated NPY and Y2 receptor overexpression

Human temporal lobe (hippocampus) tissue was obtained by surgical resections from three patients treated for intractable epilepsy at Rigshospitalet in Copenhagen, Denmark, as previously described [38, 39]. Prior to each surgery a written informed consent was obtained from all subjects. The use of resected human brain tissue and following procedures was approved by the local Ethical Committee in Copenhagen (H-2-2011-104) in accordance with the Declaration of Helsinki. The resected tissue was immediately submerged in ice-cold sucrose solution containing in mM: 200 sucrose, 21 NaHCO_3_, 10 glucose, 3 KCl, 1.25 NaH_2_PO_4_, 1.6 CaCl_2_, 2 MgCl_2_, 2 MgSO_4_ (all from Sigma-Aldrich, Sweden), bubbled with carbogen (O_2_, 95% and CO_2_, 5%) adjusted to 300–310 mOsm and 7.4 pH, and transferred from the surgical theatre to the laboratory. Time of transport of tissue from Copenhagen Hospital operating theatre to Lund laboratory was approximately 1 h. Coronal slices (250 μm thick) were cut on a Leica VT1200 vibratome in the same sucrose-solution as mentioned above. The slices were then transferred to ice-cold rinsing medium containing Hank’s balanced saline solution (HBSS) with 20 mM HEPES, 17.5 mM glucose and 0.5% penicillin/streptomycin solution (all from Life Technologies, Thermo Fisher Scientific Inc, Sweden) before placing them on cell culture inserts (Millipore, Sweden, #PICM03050) in 6-well plates with 960 μl equilibrated culturing medium. This medium contained 50% minimum essential media (MEM), 25% horse serum, 18% HBSS, and 2% B27 supplemented with 0.5% penicillin/streptomycin solution (all from Life Technologies, Thermo Fisher Scientific Inc, Sweden), glutamine 2 mM, glucose 11.8 mM, and sucrose 20 mM (all from Sigma-Aldrich, Sweden). Slices were cultured as interface cultures at 37 °C, 5% CO_2_, and ambient O_2_ in 90% humidity.

The slices were allowed to settle for 12 h before addition of the AAV1-NPY/Y2 vector (titre of 10^12^ vg/ml), with approximately 0.035 µl/mm^2^ added (3.5 * 10^7^ vg/mm^2^). After 10 days of culturing, slices for immunohistochemistry were fixed in 4% paraformaldehyde in phosphate buffer (PB) for 12–24 h, rinsed in PB and then stored until further processing in anti-freeze solution (ethylene glycol and glycerol in PB) at −20 °C.

### NPY immunohistochemistry in organotypic human hippocampal slices

Sections were fixed in 4% paraformaldehyde for 20 min. Sections were washed three times in KPBS for 10 min and preincubated in 10% Donkey serum, 1% Triton X-100 in KPBS for 1 h. After adding rabbit anti-NPY antibody (1:500, N9528, Sigma-Aldrich, DK) in 5% Donkey serum, 0.25% Triton X-100 in KPBS, sections were incubated overnight at 4 °C. Sections were washed 3 × 10 min in tKPBS, incubated for 2 h at RT with Cy3-conjugated Donkey anti-rabbit antibody (1:200, Jackson Immunoresearch USA) in 1% Donkey serum, 0.25% Triton X-100 in KPBS and washed 1 × 10 min in tKPBS and 2 × 10 min in KPBS. The slides were then mounted with DABCO (Sigma-Aldrich) and digitized images were obtained using Olympus BX61 microscope and CellSens software. Average fluorescence intensity of NPY immunoreactivity in hippocampus was evaluated using ImageJ. The experimenter performing the evaluation was unaware of the specific treatment given to individual slices.

### Quantitative PCR measurements of NPY-related genes in organotypic human hippocampal slices

Sections of organotypic slices treated with AAV1-NPY/Y2 and corresponding empty control vector were kept in −80 °C freezer until processing. The tissue was scraped from the glass sections and homogenized with QIAzol lysis reagent (QIAgen) and QIAshredder spin column (QIAgen, Denmark). Total RNA was extracted using RNeasy Lipid Tissue Kit (QIAgen) according to manufacturer’s protocol. The concentration of total RNA was measured on Nanodrop (Thermo Fisher Scientific), and the quality was evaluated by using the 260/280 nm ratio parameter. RNA (0.5 *μg*) was reverse transcribed to cDNA using High Capacity cDNA Reverse Transcription Kit (Applied Biosystems, USA) following the manufacturer’s recommendations. Quantitative PCR (qPCR) was performed using the following NPY gene primers with GAPDH as the reference gene [40].

hNPY: forward: 5’-GGA GGA CAT GGC CAG ATA CT -3’, reverse: 5’-ATC TCT GCC TGG TGA TGA GG -3’; hY2: forward: 5’-GGC CAT CTT CCG GGA GTA TT -3’, reverse: 5’-GCC AGG CCA CTT TTC AGT AC -3’; hY1: forward: 5’-AAT ACC AGC GGA TCT TCC C -3’, reverse: 5’-TTC CCT TGA ACT GAA CAA TCC -3’; hY5: forward: 5’-GCC CCG AGG TCT GCT CAT TGT -3’, reverse: 5’-TGT GGC AGG TCA GTT GTT ACC GA -3’; GAPDH: forward: 5’-TCA CCA CCA TGG AGA AGG C -3’, reverse: 5’-GCT AAG CAG TTG GTG GTG CA -3’.

The real-time qPCR was run on LightCycler480 (Roche). The cycling conditions were 1 cycle of pre-incubation at 95 °C for 5 min, followed by 45 three-segment cycles of amplification (95 °C for 10 s; 60 °C for 15 s, and 72 °C for 10 s) where fluorescence was automatically measured during PCR, and 1 cycle three-segment cycle of product melting (5 s at 95 °C, 1 min at 65 °C and then continuous acquisition mode at 97 °C for 5 acquisitions per °C). The last step was one cooling cycle for 10 s with ramp rate 2 °C/s.

### Statistics

Data analysis was done in Prism (GraphPad) and in Igor Pro (Wavemetrics). One-way ANOVA tests followed by Bonferroni’s multiple post-hoc comparison test or two-tailed Student’s t-test (one comparison) was used in the case of normally distributed data. Electrophysiology data was analysed in Igor (Wavemetrics) and Prism (GraphPad) using two-tailed Student’s paired t-test and one-way ANOVA followed by Tukey’s post hoc test. All data is presented as mean ± SEM. A difference was considered significant if *P* < 0.05.

## Results

### AAV1-NPY/Y2 treatment decreases time spent in motor seizures and increases latency to SE

Three weeks after viral vector injection, the rats were administered s.c. with KA (*n* = 77). Following the KA injection, all animals but two developed motor seizures as defined by grade 3 or above by the Racine scale (see methods section). The two animals belonged to treatment group AAV1-Y2/NPY and AAV8-NPY/Y2, respectively. One animal from the group of AAV1-EMPTY was excluded due to a failed KA s.c. injection.

**Fig. 1:**
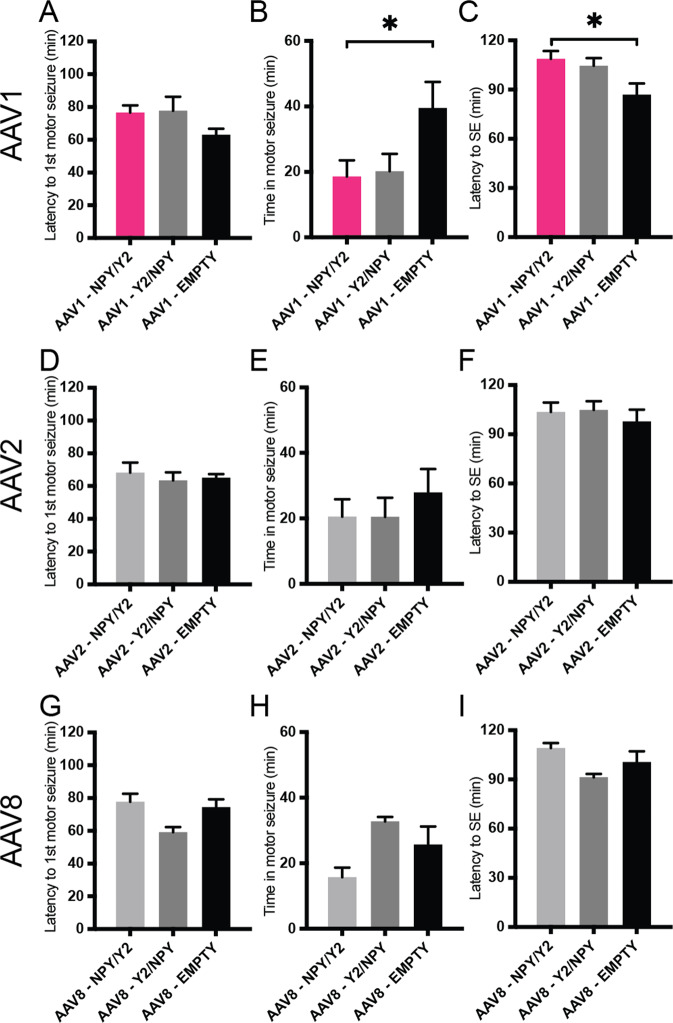
Outcomes from the seizure assessment after bilateral overexpression of the transgenes. AAV1: **A** latency to 1^st^ motor seizure, **B** time spent in motor seizures, **C** latency to status epilepticus (SE). The AAV1-NPY/Y2 vector is highlighted (magenta); AAV2: **D** latency to 1st motor seizure, **E** time spent in motor seizures, **F** latency to SE; AAV8: **G** latency to 1st motor seizure, **H** time spent in motor seizures, **I** latency to SE. Data is shown as mean ± SEM. (*n* = 7–12 in each group). *P < 0.05 versus same serotype control (EMPTY). One-way ANOVA followed by Bonferroni’s multiple comparison post-hoc tests.

Measurements of the latency until the first motor seizure (Fig. 1A, D, G) showed no significant differences between transgene groups and their respective serotype controls.

Comparing the time spent in motor seizure (Fig. 1B, E, H) revealed a statistically significant difference between the animals treated with the AAV1 serotype (one-way ANOVA, *F* = 3.6, *P* < 0.05). The AAV1-NPY/Y2 group spent less time in motor seizures (AAV1-NPY/Y2, 18.6 ± 5.0 min, *n* = 8) than the control group treated with the empty AAV1 vector (AAV1-EMPTY, 39.6 ± 7.9 min, *n* = 7, *P* = 0.049). There was no statistically significant difference between the other groups, although AAV1-Y2/NPY showed a trend towards spending less time in motor seizure (AAV1-Y2/NPY, 20.2 ± 5.3 min, *n* = 8, *P* = 0.07).

Finally, the latency to SE (Fig. 1C, F, I) in the AAV1-NPY/Y2 group was significantly longer (one-way ANOVA, *F* = 4.4, *P* < 0.05), (AAV1-NPY/Y2, 108.8 ± 4.9 min, *n* = 8) compared to the AAV1 control group (AAV1-EMPTY, 87.0 ± 6.8 min, *n* = 7, *P* = 0.02). AAV1-Y2/NPY did not differ significantly from the AAV1-EMPTY group (AAV1-Y2/NPY, 104.7 ± 4.6 min, *n* = 8, *P* = 0.07).

### NPY overexpression after AAV vector-mediated treatment

**Fig. 2:**
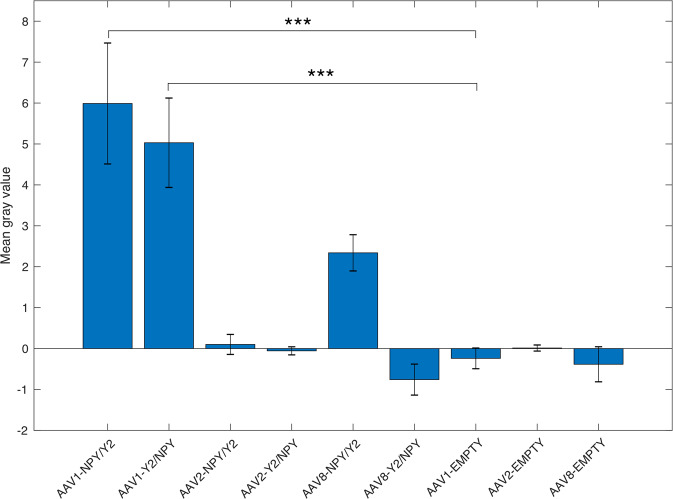
NPY expression levels after bilateral viral vector administration as measured by means of grey value in images from immunohistochemistry. Data is shown as mean ± SEM. The data was analyzed with a one-way ANOVA followed by Bonferroni’s multiple comparison test. ***P < 0.001.

To confirm successful vector delivery and NPY expression efficacy we performed immunohistochemical staining against NPY and investigated the expression levels immediately after SE (Fig. 2). Consistent with the demonstrated effect on motor seizures and latency to SE, animals injected with AAV1-NPY/Y2 vector had significantly higher levels of NPY expression (mean grey value: 6.0 ± 1.5, *n* = 8) than AAV1-Empty (mean grey value: −0.2 ± 0.3, *n* = 8, *P* < 0.001). Also, AAV1-Y2/NPY treated animals had significantly higher NPY expression levels than the controls (mean grey value: 5.0 ± 1.1, *n* = 8, *P* < 0.001). Transgene vectors with the AAV2 and AAV8 serotype, however, did not show increased expression of NPY compared to the empty control.

### Y2 receptor functional binding levels after AAV vector-mediated overexpression in rats

**Fig. 3:**
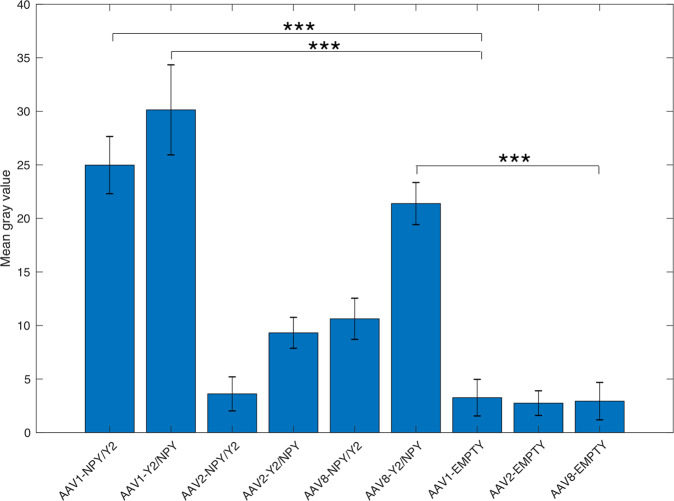
Y2 receptor functional binding intensity in the hippocampus after bilateral viral vector administration. Data is shown as mean ± SEM. Data was analysed with a one-way ANOVA followed by Bonferroni’s multiple comparison test. ***P < 0.001.

To evaluate overexpression of Y2-receptors after various viral vector treatments, a functional binding assay was performed in tissue from the dorsal hippocampus. Y2 receptor specificity was verified by assessing NPY-stimulated binding of [^35^S]-GTPγS in specimens where Y1 and Y5 receptors were preincubated and blocked with specific antagonists (BIBP3226, L-152,804) (Fig. 3). Three vectors showed higher Y2 functional binding for NPY compared to the corresponding serotype controls. AAV1-NPY/Y2 had an average mean grey value of 25.0 ± 2.7 (*n* = 8, *P* < 0.001) and AAV1-Y2/NPY (30.1 ± 4.2, *n* = 8, *P* < 0.001), which were both higher than the AAV1-EMPTY (3.3 ± 1.7, *n* = 8). Finally, the AAV8-Y2/NPY (21.4 ± 2.0, *n* = 9, *P* < 0.001) treated animals has also higher expression levels than the respective serotype control AAV8-EMPTY (1.7 ± 1.9, *n* = 8).

### Effect of AAV vectors on basal glutamatergic synaptic transmission

**Fig. 4:**
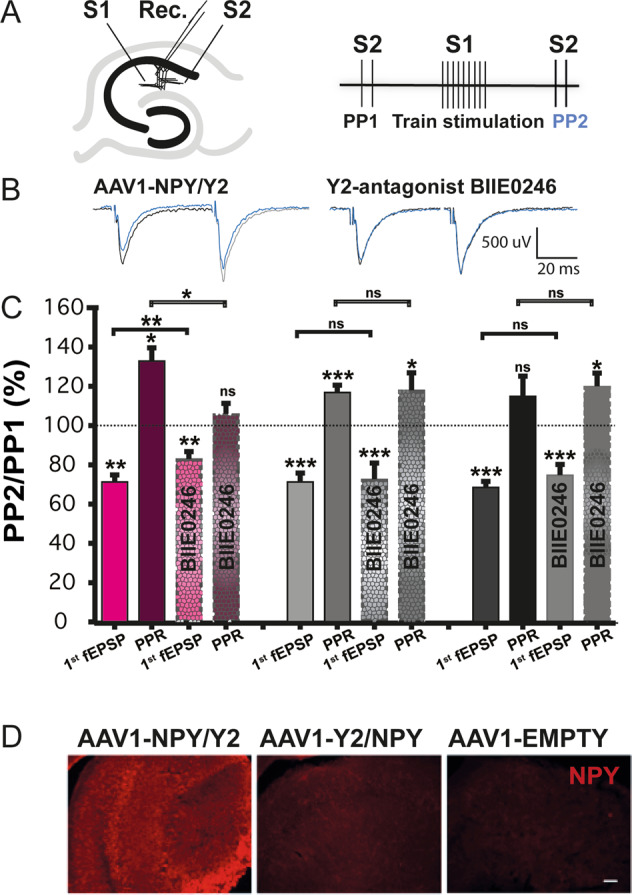
Train stimulation in slices from AAV1-NPY/Y2 treated animals decreases glutamate release in neighbouring synapses through NPY-Y2 receptor activation. **A** Experimental setup, showing placement of stimulation (S1 and S2) and recording electrodes (Rec.) in the stratum radiatum, CA1 area of the acute hippocampal slice (left) and schematic depiction of the stimulation protocol (right). The effect of transgene NPY expression on excitatory neurotransmission was also examined by applying repetitive train stimulation (TS) with 100 Hz, 10 stimulations, in one group of presynaptic fibers/pathway (S1), preceded and followed at 500 ms interval by PP-stimulation (20 Hz) in a neighbouring, convergent but independent presynaptic pathway (S2). We compared the amplitude of the fEPSPS evoked by paired-pulse stimulation (PP) before (PP1) and after train stimulation (PP2, blue **B**). Example sweeps showing fEPSP evoked by S2 before (black sweep) and after TS-stimulation (blue sweep), showing a reduction in the first fEPSP and concomitant increase in PPR after TS-stimulation. Right) This reduction is smaller in the AAV1-NPY/Y2 treated animals after application of the Y2-receptor antagonist BIIE0246. **C** Bar graph showing average depression of fEPSP after TS-stimulation in AAV1-NPY/Y2 (*n* = 5), AAV1-Y2/NPY (*n* = 7), and AAV1-EMPTY (*n* = 7). The TS-induced depression of fEPSP is reduced after application of BIIE0246 in the AAV1-NPY/Y2 treatment-group (*n* = 5), but not in AAV1-Y2/NPY (*n* = 7) or AAV1-EMPTY (*n* = 7). AAV1-NPY/Y2 (left bars), AAV1-Y2/NPY (middle bars), and AAV1-EMPTY (right bars). **D** Examples of immunohistochemical staining for NPY after electrophysiology. Scalebar 100 μm, **P < 0.01, ***P < 0.001, Student’s paired t-test. Data is shown as mean ± SEM.

Since the AAV1 serotype demonstrated a seizure-suppressant effect in animals, as evident by decreased time spent in motor seizures and increased latency to SE, we further investigated whether transgene expression also affected basal excitatory synaptic transmission, specifically glutamate release probability in transgene expressing hippocampal slices in vitro. To address this question, we used a paired-pulse stimulation protocol [41]. The relationship between two fEPSPs elicited with short inter-stimulus interval between 10 and 100 ms, a paired-pulse ratio (PPR), can typically inform whether changes in synaptic efficacy is localised pre- or postsynaptically. The PPR in CA1 stratum radiatum of AAV1-NPY/Y2, AAV1-Y2/NPY and AAV1-EMPTY were compared using different inter-stimulus intervals (ISI 25, 50, 100, and 200 ms). We could not detect any difference in baseline PPR at any stimulation interval between treatment groups (Supplementary Fig. 4). When applying the Y2 receptor antagonist BIIE0246 however, the PPR decreased significantly at the ISI 25 ms in the AAV1-NPY/Y2 treatment group, but not in AAV1-Y2/NPY (Supplementary Fig. 4), suggesting an effect of transgene NPY and Y2 on glutamate release [42, 43] at frequencies of 40 Hz (and presumably higher) in the AAV1-NPY/Y2 treatment group. To substantiate this finding, we tested if train stimulation (TS)-induced transgene NPY released from one group of glutamatergic synapses was able to diffuse to and decrease glutamate release from neighboring synapses (so-called volume transmission characteristic for neuropeptides). We gave a TS to one group of presynaptic fibers in the CA1 stratum radiatum (Fig. 4A, stimulation 1, S1) and measured the fEPSPs amplitude in a neighbouring, convergent but independent group of synapses (Fig. 4A, Stimulation 2, S2). Paired-pulse stimulations were given to S2, before and after the conditioning TS in S1 (Fig. 4B) and the fEPSP of S2 were compared before and after TS. All treatment groups showed a significant depression of the first fEPSP post-TS in the neighbouring synapses as compared to pre-TS fEPSPs (Fig. 4C). This was accompanied by an increase in PPR, suggesting that it was mediated by decreasing presynaptic glutamate release probability in all groups. The increase in PPR was higher in AAV1-NPY/Y2 treatment group, compared to both Y2/NPY and empty vector (One way ANOVA, *F* = 5.62113, Tukeys post-hoc, *p* = 0.08241, *p* = 0.00913). These results suggest different underlying mechanisms [44]. Synaptic transmitter release can impact neighbouring synapses via several mechanisms, depending on frequency of activation and time after activity [45–54]. To discern a possible contribution of NPY NPY release spillover from activate synapses in vector-treated animals, we applied the Y2 receptor antagonist BIIE0246. Y2 receptor antagonist significantly reduced post-TS fEPSP PPR only in the AAV1-NPY/Y2 group (Fig. 4C). This was accompanied by an elimination of the increase in PPR from 133.1 ± 6.6% to 106.3 ± 5.3% (*n* = 5, *P* = 0.0111, Fig. 4C) suggesting that part of the post-TS depression was mediated by transgene NPY acting on transgene Y2 receptors. Repeating this protocol in rats injected with AAV1-EMPTY and AAV1-Y2/NPY showed no such dependence on the Y2 receptor antagonist BIIE0246 and did not affect the post-TS fEPSP reduction (Fig. 4C).

### NPY and Y2 overexpression in resected hippocampal slices from human TLE patients

**Fig. 5:**
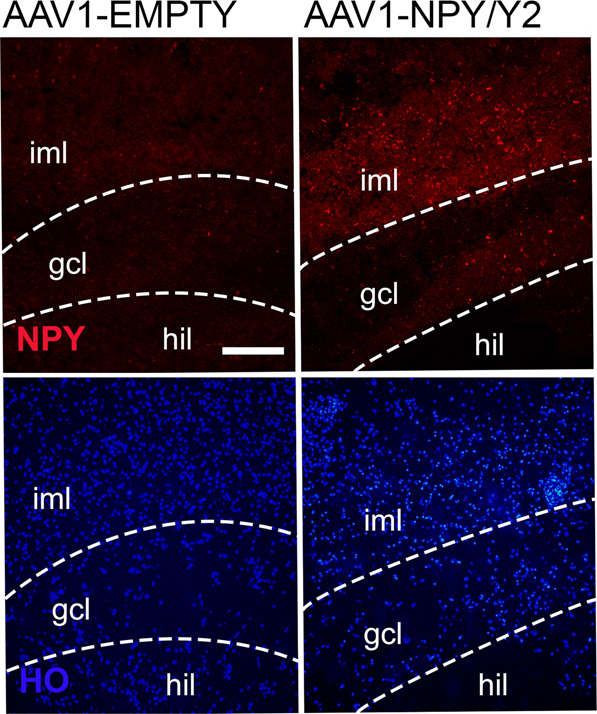
Increased NPY-immunoreactivity in AAV1-NPY/Y2 treated organotypic hippocampal slices from TLE-patients. Example images of immunohistochemical stainings of NPY in AAV1-EMPTY (left) and AAV1-NPY/Y2-treated (right) organotypic slices from one patient operated for intractable TLE. Scale bar 50 μm, iml (inner molecular layer), gcl (granule cell layer), hil (hilus), NPY (neuropeptide Y, red), HO (Hoescht stain, blue).

We have previously demonstrated that in hippocampal slices surgically removed from patients with drug-resistant TLE, NPY application decreased excitatory synaptic transmission and epileptiform activity, supporting an antiepileptic effect of NPY in human epileptic tissue [38, 39]. To further investigate translational validity of the AAV1-NPY/Y2 vector, it seemed important to demonstrate that the vector was capable of expressing the transgenes in the human tissue. To address this issue, we optimized the procedures for organotypic culturing of human hippocampal slices ex vivo in order to keep them alive and functional for several weeks that is required for the transgene expression [55]. Analysis of NPY immunohistochemistry revealed increased levels of NPY in organotypic hippocampal slices from 3 TLE patients treated with AAV1-NPY/Y2 vector as compared to AAV1-EMPTY vector (Fig. 5). The expression levels varied in slices from different patients, but on average the mean fluorescent intensity was 119% higher in AAV1-NPY/Y2-treated slices compared to controls (3 slices from each patient and treatment group, mean fluorescence for AAV1-EMPTY: 8.0 ± 1.1, *n* = 9; for AAV1-NPY/Y2: 18.3 ± 3.5, *n* = 9, *P* = 0.02, Student’s t-test).

**Fig. 6:**
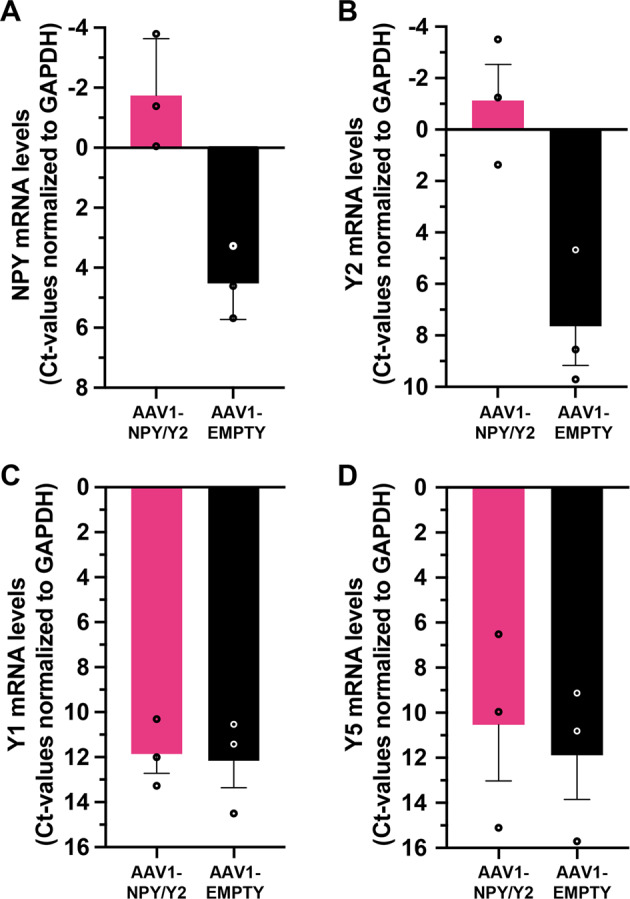
mRNA levels for NPY and Y2 are increased in AAV1-NPY/Y2-treated organotypic hippocampal slices from three TLE-patients. **A** NPY mRNA. **B** Y2 mRNA. **C** Y1. **D** Y5 mRNA levels. Data are shown as individual qPCR Ct-values normalized to GAPDH and means ± SEM. Low Ct-values (denoted as “-”) correspond to higher levels of mRNA.

Consistent with the immunohistochemistry data, measurements with qPCR demonstrated increased NPY mRNA levels in AAV1-NPY/Y2-treated compared to control vector-treated organotypic hippocampal slices from TLE-patients (Fig. 6A). Similarly, Y2 mRNA levels were also increased (Fig. 6B) while Y1 and Y5 expression did not appear to be affected in AAV1-NPY/Y2-treated slices (Fig. 6C, D).

## Discussion

This study demonstrates that the expression of combinatorial transgenes packaged in a single vector is dependent upon the serotype and the positioning of the gene sequence in the vector genome, which is especially evident in the case of AAV8. The results have important implications for clinical translation, since they underscore the significance of preclinical testing and optimization of used AAV serotype as well as gene positioning in the vector cassette. In this particular case shown here, a stronger anti-seizure effect was demonstrated for AAV1 serotype with the NPY-IRES-Y2 sequence of transgenes. In addition, we show that human epileptic tissue can be targeted by AAV1-NPY/Y2 vector to successfully mediate overexpression of NPY and Y2, strengthening the translational potential of the approach.

Based on the present study, the selection of the AAV1-NPY/Y2 vector as a priority vector was made according to a combined assessment of all modalities of outcome: seizure suppression, high overexpression of NPY and Y2 receptor, inhibition of glutamate release and finally ability to upregulate NPY and Y2 receptor in human tissue. Thus, this vector was used to demonstrate that it has a significant seizure-suppressant effect in the rat intrahippocampal KA-induced post-SE chronic model of epilepsy [26]. The latter study also confirmed a widespread and consistent expression of transgenes in the hippocampus by chosen vector, as revealed by immunohistochemistry for NPY and Y2 receptor functional binding. The preparatory investigation for vector pre-selection presented here has proven to be important for several reasons: it has been demonstrated in previous studies that the AAV serotype may contribute substantially to the tropism and spread of transgene expression when a general promoter is used [56]. Moreover, the same AAV serotype may express genes differently in different brain regions [27] and is also dependent on rodent strain [57]. In addition, the second sequential positioning combined with IRES separation may negatively affect the level of gene expression [29]. Our present data demonstrate that in the same species strain (Wistar rats), the transgene expression in the hippocampus (same brain region) significantly differed when various AAV serotypes and gene positioning were used. Importantly, we demonstrate that high levels of NPY and Y2 expression were achieved by AAV1 and AAV8, but not AAV2 vector serotype. Moreover, functional outcome, in terms of seizure suppression, was better in AAV1 vector with NPY/Y2 sequence as compared to AAV1-Y2/NPY one. Thus, the present data identify AAV1-NPY/Y2 vector as a most suitable one for further evaluation. In addition, the AAV1-NPY/Y2 vector has recently been shown to express the transgenes selectively in hippocampal neurons [40], which is the preferable tropism characteristics for intended targeting of neuronal networks.

One additional important step towards clinical application presented here is the demonstration that selected AAV vector has ability to a consistently express transgene NPY and Y2 in human hippocampal tissue resected drug-resistant TLE patients, the intended target tissue for gene therapy. An investigation of whether AAV1 can better express transgenes also in human tissue compared to the AAV2 and AAV8 serotypes would have been valuable, given variability of serotype performance reported to vary in different mouse strains [57]. However, due to the limited access to human tissue, the AAV1-NPY/Y2 vector candidate were selected for a validation of the expression in human brain slices. The results support the expectation that the AAV1-NPY/Y2 vector will express the transgenes when used in TLE patients in future clinical trials. Consistent with our finding, others have demonstrated that an AAV1 vector encoding NPY can successfully transduce human SH-SY5Y neuroblastoma cell line in vitro [58]. These data could provide a valuable step towards clinical application, demonstrating feasibility of the gene therapy approach [59].

Despite pronounced overexpression of Y2 by the AAV1-NPY/Y2 vector in organotypic human slices, there was no indication of some kind of compensatory changes in the expression of other NPY receptors Y1 and Y5. This might suggest that AAV1-NPY/Y2 will not induce a complex dysregulation of other NPY receptors which might interfere with the antiepileptic effects of the overexpressed transgenes [60–62]. Recently, it was reported that entopic overexpression of NPY in transgenic mice was associated with a mild increase in anxiety-like behaviour, which was hypothesised to be a result of receptor desensitisation [63]. It raises possibility of that also AAV derived NPY overexpression might modify NPY receptor function. However, the present approach combinatorial approach potentially overcomes such desensitisation by the concomitant expression of the Y2 receptors.

As mentioned above, we found a relatively low expression of transgenes by the AAV2 serotype resulting in corresponding functional inefficacy in suppressing stimulation-induced seizures. When used as a designated serotype, or mixed with other serotypes, AAV2 demonstrated relatively good expression of transgenes in CNS [64]. However, in studies with a direct comparison with other serotypes, the AAV2 has been reported to result in lower transgene expression in CNS, both in mice [27, 56, 65] and rats [28] relative to others. One potential explanation could be the inferior spread of the AAV2 serotype due to its interaction with heparan sulfate proteoglycan (HSPG), which acts as a co-receptor for binding the target cell membrane [66]. The HSPG is abundantly expressed in neural extracellular matrix, and might hinder diffusion of the AAV2 serotype capsids, resulting in limited expression of the transgenes [67].

We here provided evidence for better functionality of the AAV1-NPY/Y2 vector also in electrophysiological experiments in vitro. The changes in ratio of the first fEPSP amplitude to the second, PPR, together with changes in fEPSP amplitude suggests that the effect is mediated by a presynaptic mechanism, i.e., decreased transmitter release probability [41]. Thus, our findings are in good agreement with the proposed mechanism of NPY action via presynaptic Y2 receptor activation: inhibition of N- and P/Q-type voltage-dependent calcium channels (VDCC; [14]), resulting in decreased glutamate release. This was observed only in AAV1-NPY/Y2 treated animals, correlating with observed efficacy in seizure suppression by this vector. It is known that NPY is stored in large dense core vesicles (LDCVs), and presumably requires trains of repetitive activity to be released from the presynaptic terminals [12, 68, 69]. In addition, release of endogenous NPY has been shown to require activation of both temporoammonic and schaffer collateral pathways [70]. This is consistent with the lack of effect of Y2-antagonist on train-induced depression/heterosynaptic depression in slices from AAV1-EMPTY treated animals. In contrast, glutamate release probability after TS-stimulation was sensitive to Y2 antagonist after train stimulation in AAV1-NPY/Y2 slices, most likely due to a NPY spillover phenomenon [43]. Taken together, both in vivo and in vitro experiments further corroborate the potential use of this AAV1-NPY/Y2 vector for clinical gene therapy trials against focal epilepsies, such as TLE [22]. As mentioned above, the ability of this vector to express NPY and Y2 transgenes in human epileptic tissue as demonstrated here further supports this idea.

In conclusion, the present study demonstrates a necessity and importance of specific AAV serotypes in preclinical development for CNS gene therapies. In case of combinatorial gene therapy, sequence positioning in the vector plasmid has to be also addressed by evaluating functional efficacy. Importantly, transgene expression in human brain tissues also needs to be demonstrated. Finally, in more specific terms, the present study provides a number of major novel findings to be taken into account for the NPY/Y2 system as a gene therapy approach for epilepsy: (i) the identification of the most optimal serotype, AAV1 over AAV8 and AAV2; (ii) transgene sequence of the vector, NPY/Y2 over Y2/NPY; and finally the ability of this vector to express transgenes in human tissue.

## Supplementary information

Supplementory Information

## Data Availability

All raw data and materials used for figure generation in this study are available by contacting the corresponding author.
