# Supplementary material for: Combinatorial gene therapy for epilepsy: Gene sequence positioning and AAV serotype influence expression and inhibitory effect on seizures
Source: Gene Ther. 2023 Apr 7;30(7-8):649–58. doi: 10.1038/s41434-023-00399-w (PMC10457185; doi:10.1038/s41434-023-00399-w)
Supplement: Supplementary file 1 — Supplementory Information [file 41434_2023_399_MOESM1_ESM.docx]

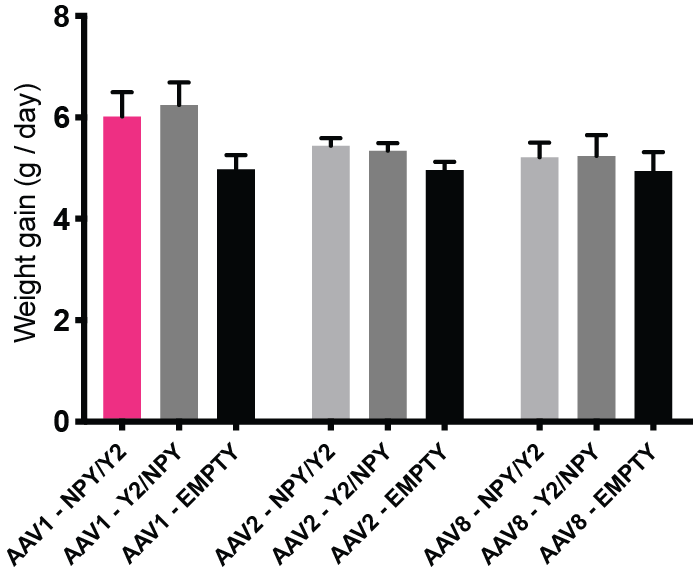


**Supplementary Figure 1**: No difference in weight gain per day since the viral vector injection was detected between the groups. Data is shown as mean ± SEM (ANOVA: F=1.92, P=0.07).


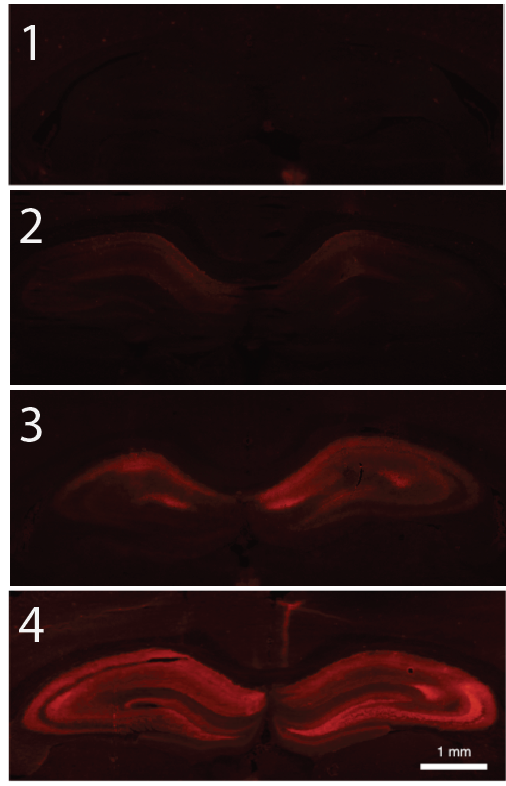


**Supplementary Figure 2:** Representative images of NPY immunohistochemistry in the dorsal hippocampus, indicating the range of signal intensity observed in the groups. Image 1 corresponds to endogenous levels, and image 4 to strong overexpression of NPY.


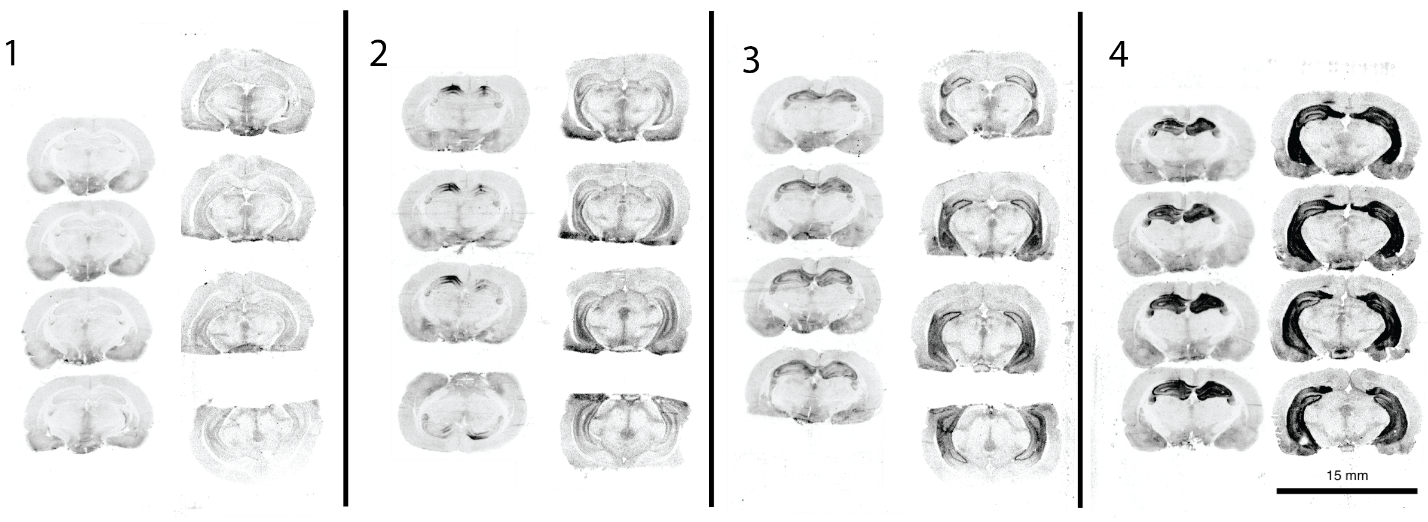


**Supplementary Figure 3:** Representative image of Y2 receptor functional binding, indicating the range of signal intensity observed in the groups. Image 1 corresponds to endogenous Y2 receptor levels, and image 4 to strong overexpression of the Y2 receptor.


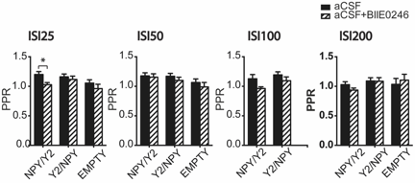


**Supplementary Figure 4:** No difference in paired-pulse ratio (PPR) was detected in either ISI between the different constructs. However, when the Y2 specific antagonist BIIE0246 was added, the AAV1-NPY/ Y2 construct resulted in a significant difference in the ISI of 25 ms. Data is shown as mean ± SEM. One-way ANOVA followed by Tukeys posthoc test (F=6.75, P=0.0460). * P<0.05.
